# The Effects of *Helicobacter pylori* Infection on Microbiota Associated With Gastric Mucosa and Immune Factors in Children

**DOI:** 10.3389/fimmu.2021.625586

**Published:** 2021-03-24

**Authors:** Wei Zheng, Jing Miao, Lingling Luo, Gao Long, Bo Chen, Xiaoli Shu, Weizhong Gu, Kerong Peng, Fubang Li, Hong Zhao, Benson O. A. Botchway, Marong Fang, Mizu Jiang

**Affiliations:** ^1^ Department of Gastroenterology, Children’s Hospital, Zhejiang University School of Medicine, National Clinical Research Center for Child Health, National Children’s Regional Medical Center, Hangzhou, China; ^2^ Department of Pathology, Children’s Hospital, Zhejiang University School of Medicine, National Clinical Research Center for Child Health, National Children’s Regional Medical Center, Hangzhou, China; ^3^ Institute of Neuroscience, Zhejiang University School of Medicine, Hangzhou, China

**Keywords:** *Helicobacter pylori*, children, gastric microbiota, 16S rRNA, RNA sequencing, immune factor

## Abstract

**Background:**

*Helicobacter pylori* infection is the main cause of chronic gastritis in children. Little is known about the effect of *Helicobacter pylori* on microbiota and immunity. This study was aimed at characterizing stomach microbiota and immune-regulatory properties of children with *Helicobacter pylori* colonization.

**Methods:**

We studied 122 children who had undergone gastric endoscopy due to gastrointestinal symptoms, 57 were diagnosed with *Helicobacter pylori* infection. Endoscopic mucosal biopsy samples were obtained for DNA and RNA extraction. Microbiomes were analyzed by 16S rRNA profiling, with the differentially expressed genes analyzed using RNA sequencing. The RNA-sequencing results of selected genes were validated by qRT-PCR.

**Results:**

Bacterial diversity of *Helicobacter pylori*-positive gastric specimens were lower than those of negative, and both groups were clearly separated according to beta diversity. *Helicobacter pylori*-positive group significantly reduced proportions of six phyla and eight genera; only *Helicobacter* taxa were more abundant in *Helicobacter pylori-*negative group. Gastric tissues RNA sequencing showed increased expression of multiple immune response genes in *Helicobacter pylori* -infection. *Helicobacter pylori* -infected children with restructured gastric microbiota had higher levels of FOXP3, IL-10, TGF-β1 and IL-17A expressions, which were consistent with increased CD4^+^T cell and macrophagocyte, compared with non-infected children.

**Conclusions:**

Presence of *Helicobacter pylori* significantly influences gastric microbiota and results in lower abundance of multiple taxonomic levels in children. Meanwhile, it affects gastric immune environment and promotes the occurrence of gastritis.

**Clinical Trial Registration:**

[http://www.chictr.org.cn], identifier [ChiCTR1800015190]

## Introduction

Prior to the discovery of *Helicobacter pylori* (*H. pylori*) in 1984 (1), the human stomach was considered to be a sterile organ (2). In recent years, culturing and high-throughput sequencing of gastric juice and biopsy have revealed a complex bacterial microbiota in the upper gastrointestinal tract (3). *H. pylori*, a Gram-negative bacterium, is usually acquired in early childhood (4), with reported links to various degrees of gastric mucosal inflammation, such as chronic gastritis, peptic ulcers and adenocarcinoma (5, 6). *H. pylori* evidently predominates in the gastric mucosa of infected individuals, while uninfected individuals exhibit a higher degree of biodiversity (7, 8). The effects of *H. pylori* abundance on other genera and their interactions in diseases are still under investigation (9, 10). Recent analysis of the gastric microbiota revealed that patients with gastric carcinoma exhibit a dysbiotic microbial community with genotoxic potential, which is distinct from that of patients with chronic gastritis, and the bacterial interactions across stages of gastric carcinogenesis were different (11, 12). Although there is a causal relationship between *H. pylori* infection and duodenal ulcer disease, few studies have reported duodenal microbiota changes caused by *H. pylori* infection (13). Minimal research studies pertaining to the presence and composition of gastric and duodenal microflora in children have been reported. With several studies elucidating the similarity between *H. pylori* burden and genotype distribution in infected children and adults (14, 15), we hypothesize that presence of *H. pylori* in children might affect microecological composition of both stomach and duodenum (16).

Emerging evidence have shown that intestinal microbiota plays a fundamental role in regulating inflammatory responses by inducing local regulation of T cell differentiation that inhibits inflammatory response (17, 18). However, the effects of commensal microbiota and early life events in the upper digestive tract on microbial composition and immune cell responses in the upper gastrointestinal mucosa have been poorly studied, especially in humans (19). *H. pylori* drives local T helper 1 cell (Th1) and T helper 17 cell(Th17 responses promote gastric mucosal inflammation (20). Also, regulatory T (Treg) cells down-regulated Th1 and Th17 immune responses and reduced inflammatory reactions to *H. pylori* infection (21). Based on the above, we hypothesize that *H. pylori* infection affects microbiota changes related to the gastric mucosa, and the changes in the microbiota affects the synthesis and secretion of Tregs, which in turn affects the local immune function and inflammatory response of gastric mucosa. To address this issue, we characterized the effect of gastric *H. pylori* colonization on local systemic immune responses and the composition of gastro-duodenal microbiota.

## Materials and Methods

### Study Cohort

**Figure 1:**
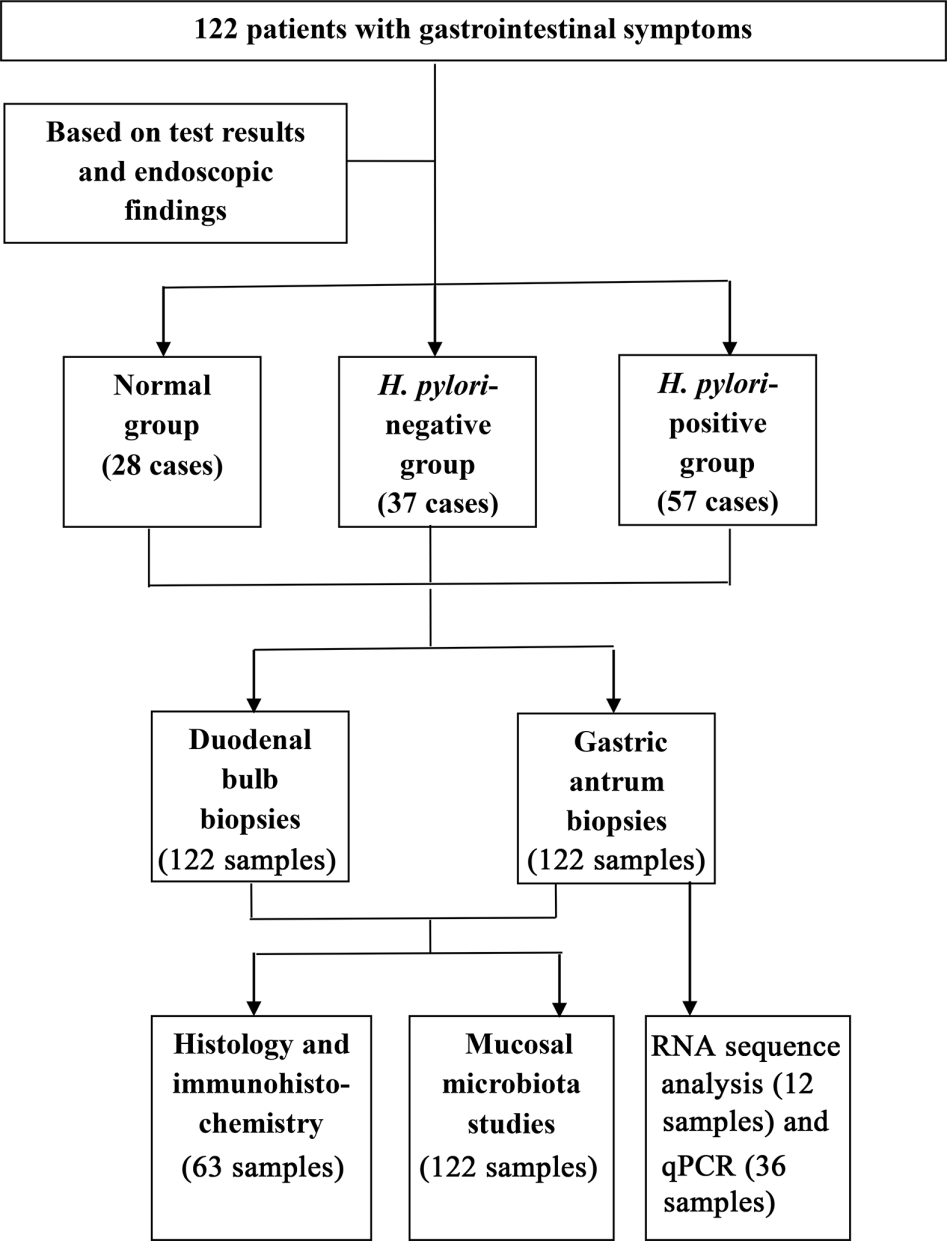
Flow diagram of the patients that were invited to participate in the study.

This study included 122 children (<16 years of age), who between January 2018 and August 2018, exhibited gastrointestinal symptoms suggestive of peptic ulcer disease, inclusive of recurrent abdominal discomfort and pain, and dyspepsia (**Figure 1**). The children, comprising of 70 (57.4%) boys and 52 (42.6%) girls, were admitted to the Children’s Hospital of Zhejiang University School of Medicine. The 122 children had a mean age of 10.2 ± 3.1 years. Exclusion criteria included a history of acute onset of symptoms, the use of antibiotic, antacid, H_2_ receptor antagonist, proton-pump inhibitor (PPI), bismuth-containing compounds, or non-steroidal anti-inflammatory drugs (NSAID) in the last 4 weeks. The study protocol was approved by the Medical Ethics Committee in the Children’s Hospital of Zhejiang University School Of Medicine (2018-IRB-004). Written informed consent was obtained from legal representatives of the children who participated in the study.

### Gastric and Duodenal Biopsies and *H. pylori* Testing

Patients underwent esophagogastroduodenoscopy at the Children’s Hospital of Zhejiang University School of Medicine. Gastric antrum biopsies were obtained for rapid urease test, culture, histology, immunohistochemistry, quantitative RT-PCR, RNA sequence analysis and gastric microbiota studies. Duodenal bulb biopsies were obtained for histology and duodenal microbiota studies. Endoscopic findings were also recorded. Gastric mucosa biopsy samples taken from the antrum were preserved in the brain-heart infusion broth (Oxoid, Dardilly, France) with 5% glycerin and sent to the laboratory of Hangzhou Zhiyuan Medical Inspection Institute. The other biopsies were frozen at -80°C until DNA and RNA extraction. The homogenate of stomach biopsy specimens was inoculated onto Columbia agar plates (Oxoid) supplemented with 5% fresh defibrinated sheep blood and kept under microaerophilic conditions (5% O_2_, 10% CO_2_, and 85% N_2_) at 37°C for 3 days. Colonies displaying typical *H. pylori* morphology were selected and identified by Gram staining and urease, oxidase, and catalase activity test. HP infection diagnosis was determined using the diagnostic criteria from the study “Consensus on diagnosis and treatment of HP infection in children” published in the Chinese Journal of Pediatrics (22). On the basis of that study, one of the following four diagnostic criteria can be used to diagnose HP infection: 1. Positive results from gastric HP bacterial culture; 2. Positive results from the pathological examination of gastric mucosa biopsy and rapid urease test (RUT); 3. In the event of inconsistencies arising between pathological examination of the gastric mucosa and RUT results, non-invasive detection, such as ^13^C urea breath test (UBT) or stool antigen test (SAT) can be performed; 4. Positive results from either pathological histology of the gastric mucosa or RUT in the event of peptic ulcer bleeding. Based on test results and endoscopic findings, patients were divided into three groups (**Table 1**): 1. *H. pylori*-positive group (HP+) (n = 57) [results consistent with *H. pylori* infection diagnosis]; 2. *H. pylori*-negative group (HP-) (n = 37) [No *H. pylori* infection, but endoscopic examination showing mucosal edema, erosion and ulcers, histopathological examination showed various degrees of inflammation]; 3. Normal group (Control) (n = 28) [No *H. pylori* infection, and no abnormal endoscopic mucosal changes and no abnormal histological examination].

**Table 1:** Demographic and clinical features of the study subjects.

Group	HP+ (*n*, %)	HP- (*n*, %)	Control (*n*, %)	*P*
Mean age				
± S.D	9.86 ± 3.09	9.72 ± 3.42	10.73 ± 2.43	0.42
Gender (number)				
Female	24 (42.11%)	17 (45.95%)	11 (39.29%)	0.86
Male	33 (57.89%)	20 (54.05%)	17 (60.71%)	
Endoscopic finding (number)				
Normal	0	0	28 (100%)	–
Esophagitis	2 (3.51%)	0	0	–
Nodular gastropathy	52 (91.23%)	27 (72.97%)	0	–
Gastric ulcer	2 (3.51%)	1 (2.70%)	0	–
Duodenal ulcer	13 (22.81%)	7 (18.92%)	0	–

### DNA Extraction

Community microbial genomic DNA was extracted from each biopsy sample using DNA MiniPrep kit (AXYGEN, China) (23). Briefly, the biopsy samples were lysed by incubating the sample in ATL lysis buffer with proteinase K overnight at 56°C and following mechanical lysis with Fast prep instrument (MP Biomedicals, Carlsbad, CA) for 1 minute at the level of 6.0 m/s, purified with spin columns, and eluted with 400 μl of buffer AE. Quality and quantity of DNA was measured with Nanodrop (Thermo Fisher Scientific, Massachusetts, USA). The extracted DNA was stored at −80°C prior to use.

### 16S rRNA Sequencing

The V3-V4 hyper-variable regions of the 16S rRNA gene were amplified using a universal primer set (341F: CCTACGGGNGGCWGCAG, 785R: GACTACHVGGGTATCTAATCC) e concentration. PCR was carried out under conditions described by Caporaso et al. (24). High fidelity DNA polymerase: TaKaRa EX Taq was used in PCR. PCRwith a barcode. All template DNAs were normalized to the sam products were separated by electrophoresis in 2% agarose gels, purified with a QIAGEN Gel Extraction Kit (QIAGEN, Germany) and pooled at equal concentrations. Sequencing libraries was generated using a TruSeqR^®^ DNA PCR-Free Sample Preparation Kit (Illumina, United States) following the manufacturer’s recommendations, and index codes were added. Library quality was assessed on the Qubit@ 2.0 Fluorometer (Thermo Scientific) and the Agilent Bioanalyzer 2100 system. The library was sequenced on an Illumina HiSeq 2500 platform (250-bp paired-end reads) at Novogene Bioinformatics Technology Co., Ltd. (Beijing, China).

### Microbiota Sequencing Data Analysis

Barcodes as well as forward and reverse primer sequences were removed and raw sequences were analyzed using the Quantitative Insight into Microbial Ecology (QIIME), version 1.9 (25). Based on distribution characteristics of low quality scores of MiSeq sequencing data, quality control of original data was executed. Chimera sequences were removed using the UCHIME algorithm to yield clean tags for further analysis (26). Sequence analysis was performed using UPARSE pipeline version 7.0.1001 (27), sequences were clustered into operational taxonomic units (OTU) assuming 97% similarity. Representative sequences for each OTU were screened for further annotation. RDP Classifier (Version 2.2) was used to annotate taxonomic information for each representative sequence based on the Green genes 97% reference data set (28). OTU abundance information was normalized using a standard sequence number corresponding to the sample with the fewest sequences. Subsequent analyses of diversity were performed based on this output-normalized data using QIIME.

### Histology and Immunohistochemistry

Histology was evaluated by 2 pathologists who were blinded to the other assays. Polymorphonuclear neutrophils (PMNs) infiltration (i.e. active gastric inflammation) and mononuclear inflammatory cells (MNCs) infiltration (i.e. chronic gastric inflammation), were assessed on a 4-grade scale (0, 1, 2, and 3, corresponding to none, mild, moderate and severe respectively), according to the updated Sydney classification (29).

Immunohistochemistry (IHC) was performed using the Bond PolymerDAB Detection Kit (Leica Microsystems, Weltzar, Germany) and Bond-X automated IHC slide staining system (Leica Microsystems). Formalin-fixed, paraffin-embedded serial sections 4-mm thick was de-paraffinized and dehydrated. The sections were heated in citrate buffer (pH 6.0, 10 mmol/L) in a microwave for 20 minutes to retrieve the antigens. Endogenous peroxidase activity was blocked with 0.3% hydrogen peroxide for 10 minutes and then incubated with primary antibodies, rabbit polyclonal anti-CD4 antibody (dilution 1:500, ab133616; Abcam, Cambridge, UK) and rabbit polyclonal anti-CD68 antibody (dilution 1:1000, ab213363; Abcam). Sections were then held in phosphate-buffered saline and incubated with horseradish-peroxidase polymer for 30 minutes, with the reaction was visualized using 3,3-diaminobenzidine tetrahydrochloride for 5 minutes and counterstained with Mayer hematoxylin. CD4 and CD68 showed cytoplasmic positive reaction. The number of positive stained cells was evaluated in 10 representative visual fields (×400, 0.0625mm^2^).

### RNA Sequencing and Data Analysis

Total RNA (6 cases in HP+ and HP-) was isolated using RNeasy mini kit (Qiagen, Germany). Paired-end libraries were synthesized by using the TruSeq™ RNA sample preparation kit (Illumina, USA) following TruSeq™ RNA Sample Preparation Guide. Briefly, the poly-A containing mRNA molecules were purified using poly-T oligo-attached magnetic beads. Following purification, the mRNA was fragmented into small pieces using divalent cations under 94°C for 8 minutes. The cleaved RNA fragments were copied into first strand cDNA using reverse transcriptase and random primers. This was followed by second strand cDNA synthesis using DNA Polymerase I and RNase H. These cDNA fragments were put through an end repair process, the addition of a single ‘A’ base, and the adapters ligated. The products were then purified and enriched with PCR to create the final cDNA library. Purified libraries were quantified by Qubit^®^ 2.0 Fluorometer (Life Technologies, USA) and validated by Agilent 2100 bioanalyzer (Agilent Technologies, USA) to confirm the insert size and calculate the mole concentration. Cluster was generated by cBot with the library diluted to 10 pM and then sequenced on the Illumina NovaSeq 6000 (Illumina, USA). Paired-end sequence files (fastq) were mapped to the reference genome using Hisat2 (Hierarchical Indexing for Spliced Alignment of Transcripts, version 2.0.5). The output SAM (sequencing alignment/map) files were converted to BAM (binary alignment/map) files and sorted using SAM tools (version 1.3.1).

### Quantitative RT-PCR Analyses for Treg Gene and Cytokine Expression

*Total RNA isolation and cDNA synthesis.* Total RNA (HP+, HP- and Control, 12 cases) was extracted and, after reverse transcription, real-time PCR was performed on duplicate cDNA samples for Forkhead box protein 3 (FOXP3), interleukin-10 (IL-10), transforming growth factor-β1 (TGF-β1) and interleukin-17A (IL-17A), and mRNA levels were analyzed by comparing the differences in fold change in mRNA normalized to glyceraldehyde 3-phosphate dehydrogenase mRNA. The primer sequences were as follows: GAPDH (forward), ACATCGCTCAGACACCATG; (reverse), TGTAGTTGAGGTCAATGAAGGG; FOXP3 (forward), GTGGCCCGGATGTGAGAAG; FOXP3 (reverse), GGAGCCCTTGTCGGATGATG; IL-10 (forward), CGAGATGCCTTCAGCAGAGT; IL-10 (reverse), CCCTTAAAGTCCTCCAGCAA; TGF-β1 (forward), AGTGGTTGAGCCGTGGAG; TGF-β1 (reverse), AGTGGTTGAGCCGTGGAG; IL-17A (forward), AGATTACTACAACCGATCCACCT; IL-17A (reverse), GGGGACAGAGTTCATGTGGTA.

*Standard curve construction.* For all cytokine and reference genes, a standard curve from serial dilutions of a known concentration of purified DNA was achieved (30). This quantified DNA consisted of the target PCR product prepared by conventional PCR from cDNA positive for the corresponding target mRNA. The copy number of each standard was calculated by standard methods using the Avogadro constant as described by Overbergh et al. (31). The log ranges of the different standard curves were from 10^7^ copies to 1 copy. Threefold measurement for each standard dilution point over the whole standard curve range was produced to generate a reliable standard curve.

*Real-time PCR and amplification protocol.* PCR amplification and analysis were achieved using a LightCycler 2.0 instrument (Roche Applied Science) with software version 4.0. All reactions were performed with the LightCycler FastStart DNA master SYBR green I (Roche Applied Science) by using a 20 μl volume in each reaction capillary. For quantification of the cytokines, 2 μl DNA standard dilutions or 2 μl cDNA was added before capillaries were capped, centrifuged, and placed in the LightCycler sample carousel. Amplification conditions consisted of an initial preincubation at 95°C for 2 min (FastStart Taq DNA polymerase activation), followed by amplification of the target DNA for 40 cycles (95°C for 15 s, 60°C for 5 s, and a variable extension time at 72°C). Melting curve analysis was performed immediately after amplification at a linear temperature transition rate of 0.1°C/s from 60 to 95°C with continuous fluorescence acquisition. Expression of FOXP3, IL-10, TGF-β1 and IL-17A mRNA relative to GAPDH mRNA were determined using the 2^-△△Ct^ method.

### Statistical Analysis

Significant differences between *a priori* predefined groups of samples were evaluated using analysis of similarity (ANOSIM) and/or permutational multivariate analysis of variance (PERMANOVA). Statistically significant differences in relative abundance of taxa were performed using linear discriminant analysis effect size (LEfSe). A significant alpha at 0.05 and an effect size threshold of 4 were used for all biomarkers discussed in this study. For the co-occurrence network analysis, OTUs with relative abundance > 0.05% of the microbiome were subjected to Spearman correlation analysis of their occurrence patterns (32, 33), using the non-rarified sequence data. Only strong correlations (*P* < 0.001 after FDR correction) were visualized through network analysis with software Cytoscape 3.6 (34). The functional genes of bacterial communities based on the 16S rRNA sequencing data was analyzed by Tax4Fun (35), with the results from Tax4Fun being further analyzed using Statistical Analysis of Metagenomics Profiles (STAMP) (version 2.1.3) (36). Differences in histological data between groups were evaluated by analysis of variance. Gene abundance in RNA-seq data was expressed as fragments per kilobase of exon per million reads mapped (FPKM). Stringtie software was used to count the fragment within each gene, and TMM algorithm was used for normalization. Differential expression analysis for mRNA was performed using R package edgeR. Differentially expressed RNAs with |log_2_(FC)| value >1 and q value <0.05, considered as significantly modulated, were retained for further analysis. Relative expression differences of FOXP3, IL-10, TGF-β1 and IL-17A mRNA were determined using multivariate analysis of variance. All tests of significance were two sides, and *P* < 0.05 was considered statistically significant.

## Results

### Comparison of Gastric and Duodenal Microbiotas

**Figure 2:**
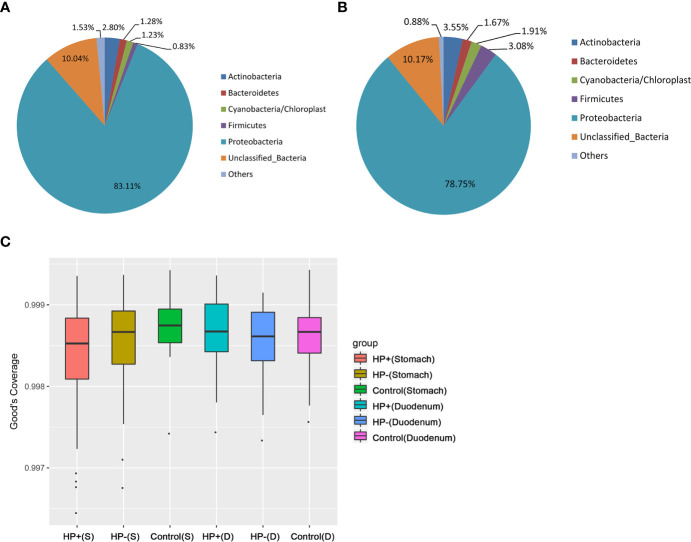
Gastric and duodenal microbiotas of children with and without *H. pylori* infection. **(A)** Average proportion of the five most abundant phyla of gastric bacteria in children (n = 122). **(B)** Average proportion of the five most abundant phyla of duodenal bacteria in children (n = 122). **(C)** Good’s coverage estimator measured the proportion of total bacterial species representative of each group’s gastric and duodenal sample.

Based on 16S rRNA bacterial gene sequencing, microbiome profiles in the gastric mucosa of 122 individuals were dominated by phyla Proteobacteria, Actinobacteria, Cyanobacteria, Bacteroidetes and Firmicutes (**Figure 2A**). These five phyla did also dominate the duodenal mucosa microbiota (**Figure 2B**). In ascertaining the reliability of bacterial diversity estimation, proportion of total bacterial species was measured using Good’s coverage estimator; the Good’s coverage index of all samples ranged from 99.6% to 99.9% (**Figure 2C**).

### The Influence of *H. pylori* Infection on the Gastric and Duodenal Microbiota

**Figure 3:**
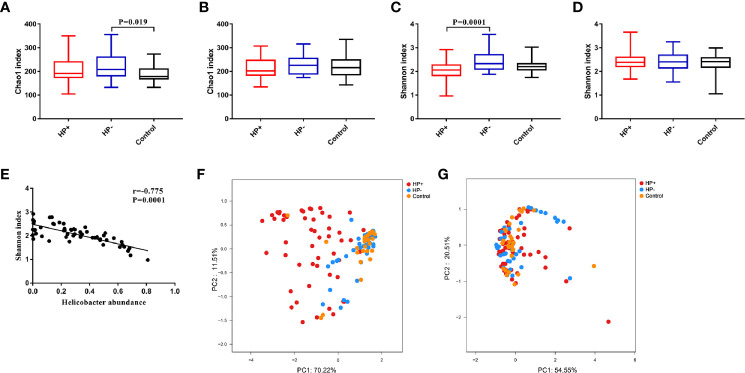
Alpha diversity and Beta diversity of microbiotas profile in children among the three groups. **(A)** Chao1 index in the stomach. **(B)** Chao1 index in the duodenum. **(C)** Shannon index in the stomach. **(D)** Shannon index in the duodenum. **(E)** Correlation between Shannon diversity and *Helicobacter* abundance from patients with *H. pylori* infection. **(F)** Principal coordinate analysis (PCoA) demonstrating clustering of patient groups in the stomach using weighted Unifrac as the distance measure and showing the percentage of diversity captured by each coordinate. **(G)** PCoA plot of duodenal microbiota among the three groups.

*Bacterial diversity and abundance in the different groups.* These indices, including a phylotype richness estimator (Chao1) and a phylotype diversity estimator (Shannon), were summarized. Chao1 analysis showed statistically significant differences between HP- and Control in the stomach (*P* = 0.019); no significant differences between HP- and Control in the duodenum were discerned (*P* = 0.44). Both gastric and duodenal microbiotas in HP+ were not significantly different from those of HP- (*P* = 0.21 and *P* = 0.16 respectively) (**Figures 3A, B**). By measuring phylotype diversity using Shannon index, we found patients in HP+ have significantly decreased gastric microbial diversity in comparison to patients in HP- (*P* = 0.0001); however, there was no statistical difference in duodenal microbial diversity between HP+ and HP- (*P* = 0.83). Both gastric and duodenal microbiotas in HP- were not significantly different from the Control (*P* = 0.07 and *P* = 0.89 respectively) (**Figures 3C, D**). For each sample in HP+, *Helicobacter* abundance and the corresponding Shannon diversity index were calculated. Linear correlation analysis indicated that phylotype diversity levels were negatively correlated with the relative abundance of *Helicobacter* genus (*r* = -0.775, *P* = 0.0001, 95% CI -0.654- -0.905) (**Figure 3E**). Beta diversity was calculated using PCoA of weighted Unifrac distances. Gastric microbiota composition in HP+ was significantly different from that of HP- (ANOSIM: *R* = 0.37, *P* = 0.001) (**Figure 3F**). Duodenal microbiota in HP+ and HP- was not significantly different (ANOSIM: *R* = 0.016, *P* = 0.236) (**Figure 3G**). Overall composition of gastric and duodenal microbiotas of HP- was similar to that of Control (ANOSIM: *R* = 0.02, *P* = 0.199 and *R* = 0.025, *P* = 0.278 respectively) (**Figures 3F, G**).

**Figure 4:**
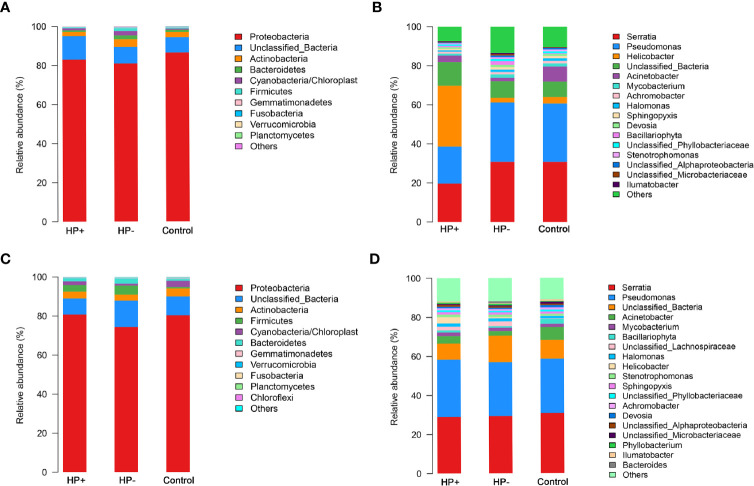
Differences of abundances in gastric and duodenal microbiota among different groups. Comparisons of the relative abundance of dominant bacterial taxa in gastric microbiota at the level of bacterial phylum **(A)** and genus **(B)**. **(C)** Relative mean abundances of phyla among the three groups in duodenum. **(D)** Relative mean abundances of genera among the three groups in duodenum.

*Comparisons between the gastric microbiota profiles in the different groups.* Overall taxonomic composition of gastric microbiota in HP+ was significantly different from that of HP- based on beta diversity. Among the major phyla of gastric microbiota (when mean abundance was greater than 0.1%), clear differences were observed between HP+ and HP-. Relative abundance of six phyla (Actinobacteria, Bacteroidetes, Firmicutes, Fusobacteria, Gemmatimonadetes and Verrucomicrobia) were significantly decreased in HP+ when compared to HP- (*P* = 0.0006 ~ 0.016). Proportions of other major phyla were not significantly different between HP+ and HP- (**Figure 4A**). At genus level, we expanded the analysis to include most abundant genera (when mean abundance was greater than 0.7% in HP+ and/or HP-). Nine genera differed in abundance between HP+ and HP-, with *Helicobacter* being more abundant in HP+ (*P* = 0.0002) and *Achromobacter, Devosia*, *Halomonas, Mycobacterium, Pseudomonas, Serratia, Sphingopyxis* and *Stenotrophomonas* being more abundant in HP- (*P* = 0.0001) (**Figure 4B**). In HP+, there was a significant difference in relative abundance of *Helicobacter* genus between individuals, with abundance levels ranging from 2.19% to 80.98% (**Table S1**). While overall composition of gastric microbiota of HP- was similar to that of Control, differences were observed between the two groups at multiple taxonomic levels. Among major phyla of gastric microbiota, three phyla differed in abundance between HP- and Control, with Cyanobacteria, Firmicutes and Proteobacteria, being more abundant in HP- (*P* = 0.043, *P* = 0.013 and *P* = 0.026 respectively) (**Figure 4A**). At the major genus level, three genera differed in abundance; relative abundance of *Bacillariophyta* was higher, and relative abundances of both *Acinetobacter* and *Helicobacter* were lower in HP- (*P* = 0.003, *P* = 0.001 and *P* = 0.001 respectively) (**Figure 4B**). Relative abundance of four phyla (Actinobacteria, Cyanobacteria, Fusobacteria and Gemmatimonadetes) were significantly decreased in HP+ group when compared to Control (*P* = 0.0008 ~ 0.03). Proportions of other major phyla were not significantly different between HP+ group and Control (**Figure 4A**). At genus level, we expanded the analysis to include most abundant genera (when mean abundance was greater than 0.7% in HP+ and/or Control). Ten genera differed in abundance between HP+ and HP-, with *Helicobacter* being more abundant in HP+ (*P* = 0.0002) and *Achromobacter, Acinetobacter, Devosia*, *Halomonas, Mycobacterium, Pseudomonas, Serratia, Sphingopyxis* and *Stenotrophomonas* being more abundant in HP- (*P* = 0.0001) (**Figure 4B**).

*Comparisons between the duodenal microbiota profiles in the different groups.* In contrast to differences in gastric microbiota, there were similar proportions of major phyla of duodenal microbiota between HP+ and HP-, only Proteobacteria was more abundant in HP+ (*P* = 0.047). Proportions of other major phyla were not significantly different between HP+ and HP- (*P* > 0.05) (**Figure 4C**). Predictably, HP+ expressed higher abundance of *Helicobacter* sequences than HP- (*P*= 0.04). With the exception of different abundances of *Helicobacter*, no statistically significant differences were observed at major genus level (**Figure 4D**). Among the major phyla in the duodenal microbiota, four phyla differed in abundance between HP- and Control, with Bacteroidetes, Cyanobacteria and Firmicutes being more abundant in HP- and Planctomycetes being more abundant in Control (*P* < 0.05, respectively). Proportions of other major phyla were not significantly different between HP- and Control (**Figure 4C**). At the major genus level, seven genera differed in abundance between HP- and Control, with *Halomonas, Stenotrophomonas* being more abundant in the HP- (*P* = 0.003 and *P* = 0.03 respectively) and *Achromobacter, Acinetobacter, Bacillariophyta* and *Devosia* being more abundant in the Control (*P* <0.01, respectively) (**Figure 4D**).

**Figure 5:**
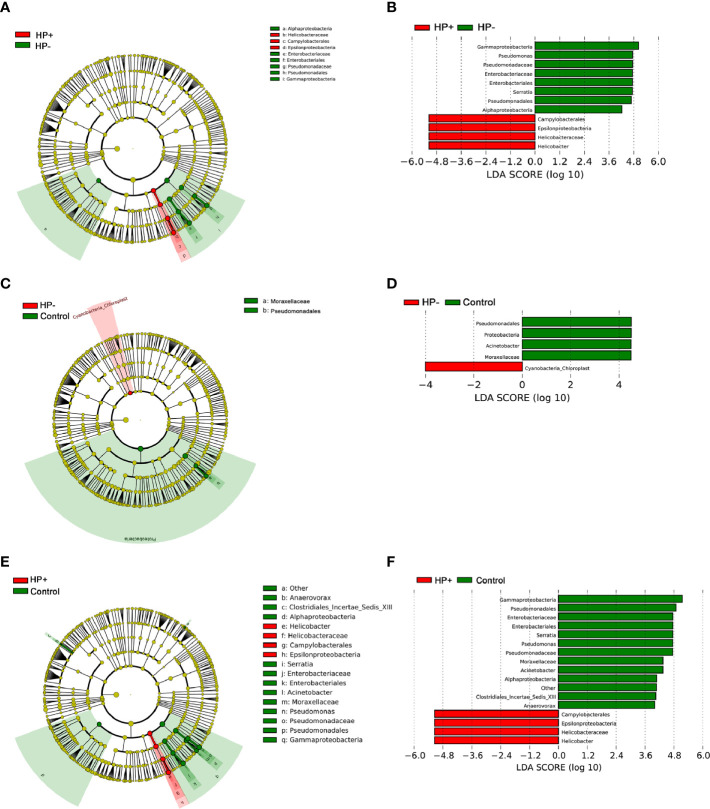
LEfSe identifies the taxa with the greatest differences in abundance among the three groups in the stomach. **(A)** The comparison between HP+ and HP-. **(C)** The comparison between HP- and Control. **(E)** The comparison between HP+ and Control. Only the taxa meeting a significant LDA threshold value of > 4 are shown. **(B)** The comparison between HP+ and HP-. **(D)** The comparison between HP- and Control. **(F)** The comparison between HP+ and Control.

To identify the most relevant taxa responsible for differences among the three groups in the stomach, we conducted LEfSe analysis (37). Taxonomic distribution was visualized using a cladogram (**Figures 5A, C, E**). This analysis identified 12 taxa including 3 genera, which were differentially abundant between HP+ and HP-. In HP+ subset, Epsilonproteobacteria (class), Campylobacterales (order), Helicobacteraceae (family) and *Helicobacter* (genus) were shown to be over-represented. In HP-, Alphaproteobacteria and Gammaproteobacteria (class), Enterobacteriales and Pseudomonadales (orders), Enterobacteriaceae and Pseudomonadaceae (families), *Pseudomonas* and *Serratia* (genera) were significantly increased (**Figure 5B**). This analysis identified 5 taxa, including 1 genus, which were differentially abundant between HP- and Control. In HP- subset, Cyanobacteria (order) were shown to be over-represented. In Control, Proteobacteria (phyla), Pseudomonadales (order), Moraxellaceae (family) and *Acinetobacter* (genus) were significantly increased (**Figure 5D**). This analysis identified 15 taxa including 4 genera, which were differentially abundant between HP+ and Control. In HP+ subset, Epsilonproteobacteria (class), Campylobacterales (order), Helicobacteraceae (family) and *Helicobacter* (genus) were shown to be over-represented. In HP- group, Alphaproteobacteria and Gammaproteobacteria (class), Enterobacteriales and Pseudomonadales (orders), Enterobacteriaceae, Moraxellaceae and Pseudomonadaceae (families), *Acinetobacter, Anaerovorax, Pseudomonas* and *Serratia* (genera) were significantly increased (**Figure 5F**).

**Figure 6:**
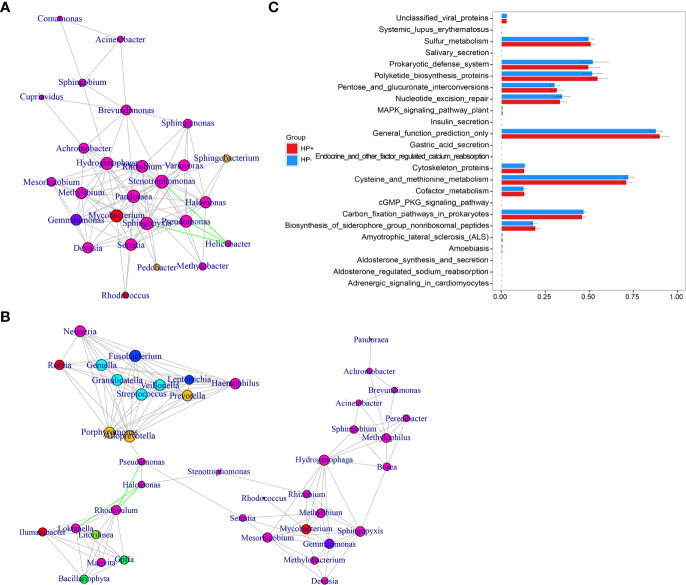
Correlation strengths of the abundant gastric microbiota and Tax4fun-based microbiome study in different stomach microhabitats. Correlation network of the abundant gastric microbiota in HP+ **(A)** and HP- **(B)**. The genera were connected (Gray: positive correlation; Green: negative correlation) when the pair-wise correlation values were significant (*P* < 0.05) after adjusting the *P* values for multiple comparisons. Furthermore, sub-community detection was performed by placing the genera in the same sub-community (represented by color of nodes) when many links were found at correlation values > 0.6 between members of the sub-community. The size of the nodes represents the degree of connections. Statistical Analysis of Metagenomics Profiles (STAMP) was used to analyze the different bacterial functions between HP+ and HP- at the KEGG level 3 **(C)**.

The overall structure of the gastric microbiotas is the result of dynamic interactions between community members. In order to detect the relationship between different members of the gastric microbial communities, we constructed a network of co-occurrence OTU and interrogated the network for modules using weighted gene co-expression network analysis (WGCNA) (**Figures 6A, B**). The correlation networks formed different bacterial clusters in the two groups, with a more complex network of interactions in HP- than that in HP+. The most dominant member, *Helicobacter*, was negatively correlated with *Pseudomonas, Sphingomonas* and *Stenotrophomonas* in HP+, those genera showed mainly positive correlations within the same bacterial clusters (**Figure 6A**). Significant negative relationships were observed between *Halomonas, Pseudomonas* and *Rhodovulum*, and there is also an obvious negative correlation between *Alloprevotella, Fusobacterium, Haemophilus, Halomonas*, *Loktanella*, *Neisseria, Porphyromonas* and *Rothia* were amongst the most well-connected and influential bacteria observed in HP- group (**Figure 6B**). The functional content of the gastric microbiota was predicted by Tax4fun based on closed-reference OTU picking. We used STAMP to analyze differences at the KEGG level 3, a database for understanding metabolic functions and at the gene level. In our present study, there were considerable gastric microbiota associated KEGG gene function changes in HP+ than HP-. At KEGG level 3 that included 396 KEGG pathways, we identified 24 different KEGG pathways that were significantly different between HP+ and HP- (**Figure 6C**). The KEGG results of the top 11 pathways enrichment are shown in **Table 2**, while 5 different KEGG pathways were significantly downregulated in HP+.

**Table 2:** The selected main microbial pathways grouped into level-3 functional categories using Tax4Fun.

KEGG Pathways	HP+	HP-	*P*
Biosynthesis of siderophore group non-ribosomal peptides	0.00195 ± 0.00022	0.00183 ± 0.00020	0.010
Carbon fixation pathways in prokaryotes	0.00461 ± 0.00015	0.00469 ± 0.00013	0.024
Cofactor metabolism	0.00133 ± 0.00006	0.00129 ± 0.00011	0.017
Cysteine and methionine metabolism	0.00712 ± 0.00028	0.00723 ± 0.00026	0.040
Cytoskeleton proteins	0.00132 ± 0.00006	0.00135 ± 0.00004	0.043
General function prediction only	0.00902 ± 0.00048	0.0088 ± 0.00037	0.027
Nucleotide excision repair	0.00336 ± 0.00038	0.00348 ± 0.00040	0.042
Pentose and glucoronate interconversions	0.00318 ± 0.00034	0.00305 ± 0.00029	0.015
Polyketide biosynthesis proteins Prokaryotic defense system	0.00549 ± 0.00053 0.00497 ± 0.00061	0.00519 ± 0.00059 0.00522 ± 0.00085	0.014 0.039
Sulfur metabolism	0.00510 ± 0.00021	0.00498 ± 0.00027	0.012

Values within a column followed by different superscript letters differ significantly (P < 0.05). Data are shown as the mean ± standard error.

### Immune Factors Associated With *H. pylori* Infection and the Gastric Microbiota

The gastric histological scores of PMN and MNC infiltrations were significantly higher in HP+ than in both HP- and Control (**Table 3**). There was no statistically significant difference in the duodenal histological scores of PMN and MNC infiltrations among the three groups.

**Table 3:** Histological scores of gastric mucosa and duodenal mucosa according to the updated Sydney classification.

Group	HP+	HP-	Control	*P*
**Stomach**				
Infiltration of PMN Infiltration of MNC	2.05 ± 0.47^#^* 0.62 ± 0.64^#^*	1.27 ± 0.46 0.18 ± 0.39	1.05 ± 0.23 0	< 0.001 < 0.001
**Duodenum**				
Infiltration of PMN	1.79 ± 0.42	1.68 ± 0.57	1.58 ± 0.65	0.387
Infiltration of MNC	0.21 ± 0.42	0.14 ± 0.35	0.11 ± 0.31	0.589

^#^HP+ vs Control P < 0.001.

*HP+ vs HP- P < 0.001.

**Figure 7:**
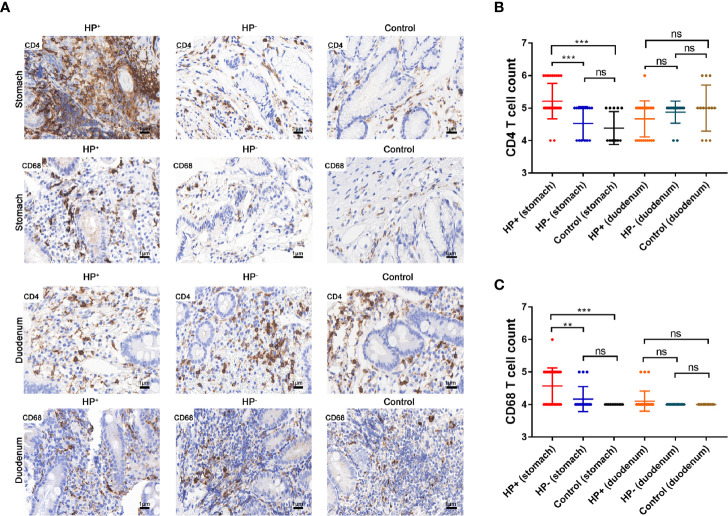
The expression of CD4^+^ T cells and CD68^+^ were assessed by immunohistochemical staining. **(A)** Immunohistochemical staining of CD4^+^ T cells and CD68^+^ in the gastric mucosa and duodenal mucosa (original magnification ×400). **(B)** Immunohistochemical score of CD4^+^ T cells. **P < 0.01, ***P < 0.001. **(C)** Immunohistochemical score of CD68^+^. **P < 0.01, ***P < 0.001.

*Relation of CD4^+^ T cells, macrophages (CD68^+^) with H. pylori infection.*The presence of CD4^+^ T cells and macrophages in the gastric mucosa were determined in two consecutive sections. IHC staining suggested that the children in HP+ (33 samples) had a significant higher number of CD4^+^ cells than the children in both HP- (17 samples) and Control (13 samples) (**Figures 7A, B**). The number of CD68**^+^** in HP+ (33 samples) was significantly higher than in both HP- (17 samples) and Control (13 samples) (**Figures 7A, C**). In the duodenal mucosa, there was no statistically significant difference in either CD4^+^ T cells or CD68**^+^** among the three groups (**Figures 7A–C**).

**Figure 8:**
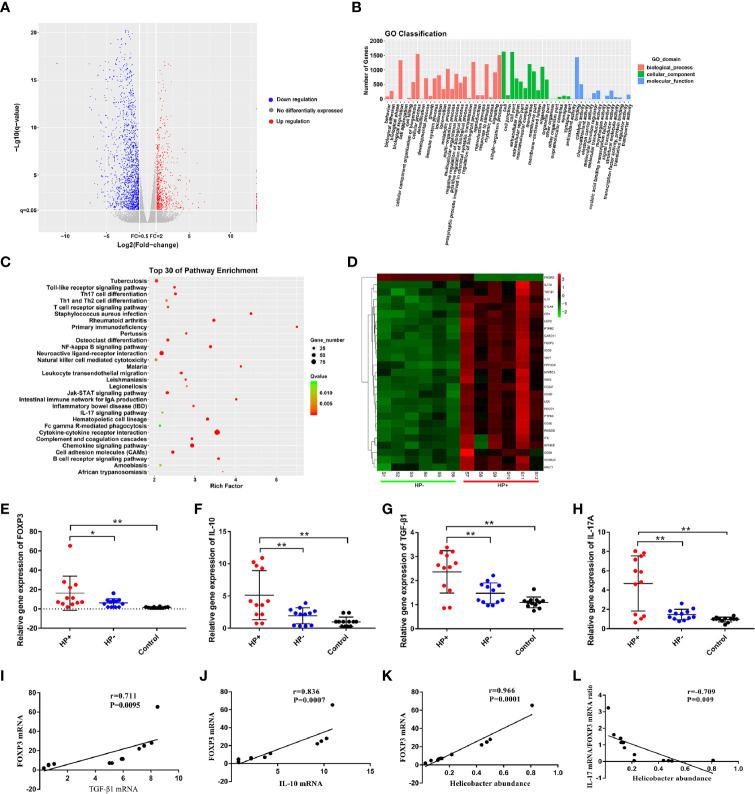
Analysis of differential gene expression using RNA sequencing, and quantitative RT-PCR validation of the selected dysregulated genes associated with T cell receptor signaling pathway, Th17 cell differentiation and TGF-β1 signaling pathway. **(A)** The volcano diagram showed significantly differentially expressed genes (DEGs) in gastric mucosa between HP+ and HP-. Red and green spots represented up-regulated and down-regulated genes respectively. Black spots indicated genes that were not differentially expressed between the two groups. **(B)** Go classification of DEGs between HP+ and HP-. The x-axis indicated the subcategories, the left y-axis represented the percentage of a specific category of DEGs and the right y-axis indicated the number of DEGs. **(C)** Scatter plot for KEGG enrichment results. The top 30 enrichment pathways are shown in the senior bubble chart. The Rich factor is the ratio of DEGs numbers annotated in this pathway term to all gene numbers annotated in this pathway term. **(D)** Cluster analysis of DEGs annotated in pathways associated with T cell receptor signaling pathway, Th17 cell differentiation and TGF-β1 signaling pathway. Samples S1 to S6 were in HP- and S7 to S12 were in HP*+*. The expression levels of FOXP3 **(E)**, IL-10 **(F)**, TGF-β1 **(G)** and IL-17A **(H)** were validated using qRT-PCR. The bar graph shows the expression of each gene in one group relative to the average expression levels in the other group. *P < 0.05; **P < 0.01, indicate a statistically significant difference. FOXP3 gene expression in relation to the regulatory cytokines gene expression and *Helicobacter* abundance level. **(I)** FOXP3 versus TGF-β1 gene expression; **(J)** FOXP3 versus IL-10 gene expression; **(K)** FOXP3 gene expression versus *Helicobacter* abundance level; **(L)** The ratio of IL-17A mRNA/FOXP3 mRNA versus *Helicobacter* abundance level.

*Analysis of differential gene expression of H. pylori infection:* The volcano diagram results showed significant differentially expressed genes (DEGs) in the gastric mucosa between HP+ and HP-. Out of the 1011 dysregulated genes detected in HP+, 180 genes were upregulated (fold change ≥ 2) and 831 genes were downregulated (fold change ≥ 2) (**Figure 8A**). *Go classification of DEGs:* To determine the function of differentially expressed genes, all DEGs were mapped to terms in the GO database. Differentially expressed mRNA was clustered in 57 GO terms. The top 10 GO terms for aggregation of differentially expressed mRNA were cell, cell composition, cellular process, single-organism process, binding, biological regulation, biological process regulation, cell membrane, response to stimulation and organelle (**Figure 8B**). *KEGG pathway analysis of DEGs between* HP+ and HP-: In order to explore the mechanism of *H. pylori* infection, we performed KEGG pathway analysis of the dysregulated genes in the gastric mucosa between HP+ and HP-. The results indicated that Th17, Th1 and Th2 cell differentiation, T cell receptor signaling pathway, Cytokine-cytokine receptor interaction, TGF-β1 signaling pathway may be involved in the pathogenesis of *H. pylori* infection, so we choose these pathways to further analyze. The KEGG results of the top 30 pathways enrichment are shown in **Figure 8C**. *Gene expression cluster:* A hierarchical cluster of DEGs is partially shown in **Figure 8D**. Genes involved in the T cell receptor signaling pathway (IL-10), Th17 cell differentiation (IL-17A, FOXP3), and TGF-β1 signaling pathway (TGF-β1) were significantly upregulated in HP+ when compared to HP-.

The RNA sequencing results of selected genes were validated by real-time RT-PCR. The dysregulated genes, FOXP3, IL-10, TGF-β1 and IL-17A in mRNA expression profiling results were selected for qRT-PCR validation. The qRT-PCR results showed that the expression of FOXP3, IL-10, TGF-β1 and IL-17A were increased in the gastric mucosa of HP+ than in both HP- and Control (**Figure 8E-H**). In HP+, FOXP3 levels were positively correlated with TGF-β1 (*r* = 0.711, *P* = 0.0095, 95% CI 0.788- 0.994; **Figure 8I**) and IL-10 levels (*r* = 0.836, *P* = 0.0007, 95% CI 0.650- 0.929; **Figure 8J**). Also, a significant correlation was identified between FOXP3 level and *Helicobacter* abundance level (*r* = 0.966, *P* = 0.0001, 95% CI 0.616- 0.974; **Figure 8K**). To determine whether *H. pylori* infection affected Treg and Th17 cells, the correlation between IL-17 mRNA/FOXP3 mRNA ratio and *Helicobacter* abundance level was evaluated; the ratio was inversely associated with *Helicobacter* abundance level (*r* = -0.709, *P* = 0.009, 95% CI -0.600- -0.930; **Figure 8L**).

## Discussion

Despite numerous studies addressing the microbiota of upper gastrointestinal tract (38), these studies are usually performed on a small number of adults infected with *H. pylori*. Information pertaining to gastric mucosal samples in children infected with *H. pylori* is scarce. Microbiota analyses show complexity and richness of the human gastric and duodenal bacterial community (39, 40), with the most abundant phyla being Proteobacteria, Firmicutes, Bacteroidetes, Fusobacteria and Actinobacteria (41, 42). In this study, we found a similar pattern. Phylum Proteobacteria was the dominant bacterial group in both stomach and duodenum irrespective of *H. pylori* infection, with a relative abundance of more than 80.00%. This was much higher than the relative abundance that had been reported in previous studies (42, 43). Studies have also reported *Streptococcus, Lactobacillus, Bacteroides, Staphylococcus, Prevotella, Fusobacterium* and *Veillonella* to be the prominent genera in the stomach (40, 44). We found a considerable higher concentration of mucosal samples belonging to *Serratia, Pseudomonas, Helicobacter*, *Acinetobacter, Achromobacter* and *Mycobacterium*. The distribution of gastro-duodenal microbiota was different from previous reports (40, 41, 44, 45). We believe the underlying reason had to do with various factors such as age, genetics, region and dietary habit.

At genus level, there was a significant change in microbial community of gastric microbiota between HP+ and HP-. In HP*+*, we found differences of relative abundance of *Helicobacter* genus between individuals in HP+ to be significant (from 2.19% to 80.98%) in the stomach. The samples with low abundance of *Helicobacter* genus could have been due to either a recent colonization with *H. pylori* or the children being colonized by a less virulent strain that was not able to dominate the ecosystem (43). Also, *H. pylori* DNA was present in the biopsy samples of subjects that were considered to be *H. pylori*-negative by conventional methods. It is possible that the number of *H. pylori* bacteria in these samples (HP- and Control) were too low to be detected by conventional methods and too few to elicit host antibody production. It could have also been due to the high sensitivity of 16SrRNA high-throughput sequencing. Although a recent study reported the influence of *Helicobacter* on the community to be more evident in duodenal samples of adults (41, 45), we, however, studied the duodenal bulb mucosal microbiota in children and ended up with a contrasting inference in our present study.

The present study indicates an inverse relationship between microbial diversity and *Helicobacter* abundance as well as the probable modulation of microbial abundance by *Helicobacter* (7, 9). Co-occurrence networks indicated that there were clear negative relationships between *Helicobacter* and the other genera. The increase in bacterial Shannon diversity among *H. pylori*-negative individuals has been previously described and would match with the study in which it was reported that higher microbiota diversity was associated with better health (46). That said, Tax4Fun analysis indicated that a variety of metabolic pathways were affected by the HP infection; the lower metabolic pathways might be associated with worse health.

Studies have reported that gastric epithelial cells and dendritic cells in the subepithlial space are localized to monitor the gastric microbiota (47–49). In mice, certain members of the gut microbiota and their metabolites have been shown to induce Treg responses in the lower intestinal mucosa. Studies have implicated Tregs to be associated with reduced Th1 and Th17 mediated gastritis in humans (14, 15). Colonization of *Bacteroides fragilis*, a prominent human commensal, was shown to induce Tregs and production of IL-10 by polysaccharide A *via* TLR2 signaling (50, 51). Clusters IV and XIVa of the genus *Clostridium* promoted Treg cell accumulation, colonization of GF mice by 46 strains of *Clostridium* provided an environment rich in TGF-β and affected Foxp3+ Treg number and function in the colon (52). Studies have demonstrated that development of Th17 cells in the small intestine can be potently induced by colonization of commensal Clostridia-related segmented filamentous bacteria (SFB) (53–55), a cascade of events initiated by SFB adhesion to the small intestine epithelium to promote IL-17 expression in RORγt^+^ CD4^+^ T cells in the gut (56). However, the role of microbiota in inducing Treg in the upper digestive tract has received little investigative attention, particularly in humans (42). Our results suggest that there is a significant correlation between *H. pylori* infection, gastric microbiota and the Treg response. Treg cells and Th17 cells are functionally antagonistic to each other, in host tolerance or defense against pathogens, especially on mucosal surfaces (57). The balance of Treg and Th17 cells play an important role in the persistence of *H. pylori* and related diseases (58). We found that FOXP3 mRNA was associated with *Helicobacter* abundance level; IL-17A mRNA/FOXP3 mRNA ratio was inversely correlated with *Helicobacter* abundance level. These indicate that the balance between Treg and Th17 cells may be biased towards Treg, which is beneficial to the persistence of bacteria, leading to chronic active gastritis. Predicted functional changes in gastric microbial communities identified alpha Linolenic acid metabolism and Arachidonic acid metabolism to be significantly upregulated, with cGMP signaling pathway and cAMP signaling pathway being significantly downregulated at KEGG level 3 in *H. pylori*-infected children. Thus, the gastric microbiota in *H. pylori*-infected children might generate short chain fatty acids and small molecules capable of modulating mucosa Treg responses (59).

It is worth noting that this study had limitations. Firstly, while it has been reported that the gastrointestinal microbiota is dynamic and can be influenced by many external factors such as drug or diet (4), we excluded subjects with recent intake of antibiotic, PPI and NSAID. Also, all biopsies were taken at fasting state during endoscopy so as to minimize the potential influence by meal. Secondly, as all cases were selected from Children’s Hospital of Zhejiang University School of Medicine, there is the possibility of results having confounding variables that are inevitable in all single-centered studies. In view of that, result from large-scale multicenter prospective clinical studies is recommended.

In summary, we report the gastric microbiota and an increased gastric Treg response in children with *H. pylori* infection. The gastric microbiota can down-regulate gastric inflammation in *H. pylori* infected children by inducing Treg responses. Our results present a comprehensive and novel perspective on microbial communities of gastric and duodenal ecosystem, which is of great significance to further study *H. pylori* colonization *in-vivo*.

## Data Availability Statement

The datasets presented in this study can be found in online repositories. The names of the repository/repositories and accession number(s) can be found below: https://www.ncbi.nlm.nih.gov/sra/PRJNA680429.

## Ethics Statement

The study protocol was approved by the Medical Ethics Committee in the Children’s Hospital of Zhejiang University School Of Medicine (2018-IRB-004). Written informed consent to participate in this study was provided by the participants’ legal guardian/next of kin.

## Author Contributions

WZ and MJ designed the study. WZ, KP, FL, and HZ collected samples. LL, GL, and BC facilitated DNA sequencing. JM and XS facilitated RNA sequencing. WZ and WG facilitated Histology and Immunohistochemistry. WZ prepared the manuscript. BB, MF, and MJ reviewed and edited the final version. All authors contributed to the article and approved the submitted version.

## Funding

This work was supported by grants from the National Natural Science Foundation of China (No. 81270459), the Public Welfare Project of Science Technology Department of Zhejiang Province (No. 2016C33152), the Scientific Research Fund of National Health and the Family Planning Commission-Major Science and Technology Project of the Zhejiang Province Medical and Health (No. WKJ-ZJ-1622), and Key Research and Development Project of Zhejiang Province (No. 2021C03064).

## Conflict of Interest

The authors declare that the research was conducted in the absence of any commercial or financial relationships that could be construed as a potential conflict of interest.
