# Supplementary material for: The Effects of Helicobacter pylori Infection on Microbiota Associated With Gastric Mucosa and Immune Factors in Children
Source: Front Immunol. 2021 Mar 24;12:625586. doi: 10.3389/fimmu.2021.625586 (PMC8024644; doi:10.3389/fimmu.2021.625586)
Supplement: Supplementary file 1 [file Table_1.docx]

**Table S1 The results of *Helicobacter pylori* (*H. polyri*) deteted by different methods and** *H.polyri* abundance analyzed by 16S rRNA profiling **of each patient in the three groups.**

| Group | Patient No. | *H. pylori* Culture | RUT | H. polyri histopathological staining | UBT | *H.polyri* abundance |
| --- | --- | --- | --- | --- | --- | --- |
| HP+ group | 1 | + | + | + | / | 37.00% |
|  | 5 | + | + | + | / | 57.00% |
|  | 7 | + | + | - | / | 14.38% |
|  | 8 | + | + | + | / | 63.22% |
|  | 9 | + | - | + | / | 80.99% |
|  | 12 | + | + | + | / | 68.00% |
|  | 13 | + | + | + | / | 62.53% |
|  | 14 | + | + | + | / | 13.48% |
|  | 15 | + | + | + | / | 45.32% |
|  | 16 | + | + | + | / | 12.03% |
|  | 17 | + | - | - | / | 2.19% |
|  | 18 | + | + | + | / | 34.71% |
|  | 19 | + | + | + | / | 6.55% |
|  | 22 | + | - | + | - | 29.68% |
|  | 24 | + | + | + | + | 51.03% |
|  | 27 | + | - | - | / | 47.54% |
|  | 28 | + | + | + | + | 28.66% |
|  | 30 | + | + | + | / | 12.30% |
|  | 31 | + | - | - | + | 64.44% |
|  | 35 | + | - | - | + | 38.10% |
|  | 37 | + | + | + | / | 42.09% |
|  | 40 | + | + | + | + | 49.66% |
|  | 41 | + | - | - | / | 67.15% |
|  | 44 | + | + | + | / | 22.61% |
|  | 47 | + | + | + | + | 50.10% |
|  | 52 | + | + | + | + | 24.49% |
|  | 53 | - | + | + | + | 68.80% |
|  | 54 | + | + | + | + | 51.98% |
|  | 57 | + | - | - | / | 11.65% |
|  | 58 | + | + | + | / | 33.83% |
|  | 60 | + | + | + | + | 15.11% |
|  | 61 | + | + | + | + | 47.46% |
|  | 65 | + | + | + | + | 14.41% |
|  | 66 | - | + | + | + | 21.73% |
|  | 67 | + | + | + | + | 22.39% |
|  | 69 | + | + | + | + | 61.38% |
|  | 70 | + | + | + | + | 67.53% |
|  | 73 | + | + | + | + | 33.45% |
|  | 79 | + | + | + | / | 27.15% |
|  | 84 | + | + | + | + | 33.68% |
|  | 88 | + | - | - | / | 4.60% |
|  | 90 | - | + | + | + | 33.84% |
|  | 96 | + | + | - | + | 6.31% |
|  | 100 | + | + | + | + | 43.84% |
|  | 101 | - | + | + | + | 15.65% |
|  | 104 | + | + | + | / | 18.73% |
|  | 105 | + | + | + | + | 28.86% |
|  | 106 | + | - | - | / | 9.59% |
|  | 107 | + | + | + | + | 23.54% |
|  | 108 | + | - | - | / | 4.12% |
|  | 111 | + | - | - | / | 26.86% |
|  | 112 | + | + | - | + | 21.95% |
|  | 113 | + | + | + | + | 29.54% |
|  | 114 | + | + | + | / | 25.87% |
|  | 115 | + | + | + | / | 11.57% |
|  | 116 | + | - | - | + | 10.34% |
|  | 122 | + | + | + | + | 10.36% |
| HP- group | 2 | - | - | - | / | - |
|  | 3 | - | - | - | / | 0.78% |
|  | 4 | - | - | - | / | 1.46% |
|  | 6 | - | - | - | / | 0 |
|  | 10 | - | - | - | / | 0.64% |
|  | 11 | - | - | - | / | 1.50% |
|  | 20 | - | - | - | / | 0 |
|  | 21 | - | - | - | / | 0.42% |
|  | 25 | - | - | - | / | 2.01% |
|  | 26 | - | - | - | / | 0 |
|  | 32 | - | - | - | / | 1.81% |
|  | 33 | - | - | - | / | 0 |
|  | 34 | - | - | - | / | 0.78% |
|  | 36 | - | - | - | / | 0.27% |
|  | 38 | - | - | - | + | 1.90% |
|  | 39 | - | - | - | / | 0 |
|  | 42 | - | - | - | / | 0 |
|  | 43 | - | - | - | / | 0.95% |
|  | 46 | - | + | - | - | 2.18% |
|  | 48 | - | - | - | / | 0 |
|  | 49 | - | - | - | / | 0 |
|  | 50 | - | - | - | / | 0 |
|  | 51 | - | + | - | - | 0.64% |
|  | 55 | - | - | - | / | 0 |
|  | 56 | - | - | - | / | 0.80% |
|  | 59 | - | - | - | / | 1.23% |
|  | 71 | - | - | - | + | 0 |
|  | 74 | - | - | - | / | 0 |
|  | 77 | - | - | - | / | 2.39% |
|  | 78 | - | - | - | / | 0 |
|  | 80 | - | - | - | / | 0 |
|  | 87 | - | - | - | / | 0.08% |
|  | 89 | - | - | - | / | 0 |
|  | 103 | - | - | - | / | 0 |
|  | 109 | - | - | - | / | 0 |
|  | 110 | - | + | - | - | 0.33% |
|  | 121 | - | - | - | + | 0 |
| Control group | 23 | - | - | - | / | 2.25% |
|  | 29 | - | - | - | / | 0 |
|  | 45 | - | - | - | / | 0 |
|  | 62 | - | - | - | / | 0.74% |
|  | 63 | - | - | - | / | 0 |
|  | 64 | - | - | - | / | 0.55% |
|  | 68 | - | - | - | / | 2.24% |
|  | 72 | - | - |  | / | 0 |
|  | 75 | - | - | - | / | 0 |
|  | 76 | - | - | - | / | 0.19% |
|  | 81 | - | - | - | / | 0 |
|  | 82 | - | - | - | / | 0.08% |
|  | 83 | - | - | - | / | 0 |
|  | 85 | - | - | - | - | 2.50% |
|  | 86 | - | - | - | / | 0 |
|  | 91 | - | - | - | / | 0.30% |
|  | 92 | - | - | - | / | 0.13% |
|  | 93 | - | - | - | / | 0 |
|  | 94 | - | - | - | / | 0.13% |
|  | 95 | - | - | - | / | 0.07% |
|  | 97 | - | - | - | / | 0 |
|  | 98 | - | - | - | / | 0 |
|  | 99 | - | - | - | / | 0 |
|  | 102 | - | - | - | / | 0 |
|  | 117 | - | - | - | / | 0 |
|  | 118 | - | - | - | - | 2.05% |
|  | 119 | - | - | - | / | 0.05% |
|  | 120 | - | - | - | / | 0 |

Note: RUT, rapid urease test; UBT, urea breath test；+，Positive; -，Negative; /, no detection;
